# *Clostridium perfringens* epsilon toxin mutant Y30A-Y196A as a recombinant vaccine candidate against enterotoxemia

**DOI:** 10.1016/j.vaccine.2014.03.079

**Published:** 2014-05-13

**Authors:** Monika Bokori-Brown, Charlotte A. Hall, Charlotte Vance, Sérgio P. Fernandes da Costa, Christos G. Savva, Claire E. Naylor, Ambrose R. Cole, Ajit K. Basak, David S. Moss, Richard W. Titball

**Affiliations:** a College of Life and Environmental Sciences, University of Exeter, Stocker Road, Exeter EX4 4QD, United Kingdom; b Department of Biological Sciences, Birkbeck College, Malet Street, London WC1E 7HX, United Kingdom

**Keywords:** *Clostridium perfringens*, Epsilon toxin pore-forming toxin, Enterotoxemia, Recombinant vaccine

## Abstract

Epsilon toxin (Etx) is a β-pore-forming toxin produced by *Clostridium perfringens* toxinotypes B and D and plays a key role in the pathogenesis of enterotoxemia, a severe, often fatal disease of ruminants that causes significant economic losses to the farming industry worldwide. This study aimed to determine the potential of a site-directed mutant of Etx (Y30A-Y196A) to be exploited as a recombinant vaccine against enterotoxemia. Replacement of Y30 and Y196 with alanine generated a stable variant of Etx with significantly reduced cell binding and cytotoxic activities in MDCK.2 cells relative to wild type toxin (>430-fold increase in CT_50_) and Y30A-Y196A was inactive in mice after intraperitoneal administration of trypsin activated toxin at 1000× the expected LD_50_ dose of trypsin activated wild type toxin. Moreover, polyclonal antibody raised in rabbits against Y30A-Y196A provided protection against wild type toxin in an *in vitro* neutralisation assay. These data suggest that Y30A-Y196A mutant could form the basis of an improved recombinant vaccine against enterotoxemia.

## Highlights

• Etx mutant Y30A-Y196A showed markedly reduced cytotoxicity towards MDCK.2 cells.
• Y30A-Y196A is inactive in mice after intraperitoneal administration.
• Y30A-Y196A is able to induce a specific antibody response in rabbits.
• Y30A-Y196A polyclonal antibody is able to induce protective immunity *in vitro*.
• Y30A-Y196A could form the basis of a recombinant vaccine against enterotoxemia.

## 1 Introduction

*Clostridium perfringens* is a Gram positive, anaerobe, spore forming bacterium that is classified into five toxinotypes based on production of the four typing toxins (α-, β-, ɛ-, and ι-toxins) [1]. Epsilon toxin (Etx), a β-pore-forming toxin, is produced by *C. perfringens* strains that belong to toxinotypes B and D and plays a key role in the pathogenesis of enterotoxemia, a severe gastro-intestinal disease of ruminants that causes severe economic losses to farmers worldwide.

*C. perfringens* toxinotype B is the etiologic agent of dysentery in newborn lambs and haemorrhagic enteritis and enterotoxemia in goats, calves and foals [2,3]. More recently, toxinotype B has been detected in a human with a clinical presentation of multiple sclerosis, providing clues for environment triggers of the disease [4]. *C. perfringens* toxinotype D affects mainly sheep and lambs but also causes infections in goats and calves [2,3].

The most important factor in initiating disease is the disruption of the microbial balance in the gut due to overeating carbohydrate rich food, which causes proliferation of *C. perfringens* and consequent overproduction of the toxin [2,5]. Overproduction of Etx causes increased intestinal permeability, facilitating entry of the toxin into the bloodstream and its spread into various organs, including the brain, lungs and kidneys. While infection of the central nervous system results in neurological disorders, the fatal effects on the organs often lead to sudden death [6,7].

For full activity of the toxin, proteolytic processing is required, with carboxy-terminal and amino-terminal peptides removed. Toxin activation typically occurs in the gut either by digestive proteases of the host, such as trypsin and chymotrypsin [8], or by λ-protease produced by *C. perfringens* itself [9,10].

To prevent Etx-induced enterotoxemia in domesticated livestock, a number of commercial vaccines are available that have been used extensively over the past decades. These vaccines are based on either formaldehyde treated *C. perfringens* type D culture filtrate or formaldehyde-inactivated recombinant wild type toxin [11,12]. These vaccine preparations have several disadvantages: (1) complete removal of free formaldehyde is required to avoid possible toxic side effects, (2) toxoiding using formaldehyde can show considerable batch to batch variation in immunogenicity of these vaccines [12], (3) inflammatory responses following vaccination can lead to reduced feed consumption [13] and (4) reversion to toxicity may occur in incompletely inactivated bacterial toxins. Therefore, there is a need to identify Etx variants with reduced toxicity relative to wild type toxin. One approach to solving this problem is to develop recombinant vaccines based on site-directed mutants with markedly reduced toxicity.

Amino acid residues Y30 and Y196 have previously been identified to play key roles in cell binding and thus, cytotoxicity of Etx [14,15]. Therefore, this study aimed to determine the potential of a site-directed mutant of Etx with mutations Y30A and Y196A combined, termed Y30A-Y196A, to be exploited as a recombinant vaccine against enterotoxemia.

## 2 Materials and methods

### 2.1 Expression and purification of recombinant Etx mutant Y30A-Y196A

The gene encoding epsilon prototoxin, *etxD*, from *C. perfringens* Type D strain NCTC 8346 was cloned into the expression vector pET-26b(+) (Merck, Darmstadt, Germany) with a N-terminal PelB leader peptide in place of the 13 amino acids N-terminal peptide sequence (residues KEISNTVSNEMSK) and with a C-terminal polyhistidine (6× His) tag as described previously [14].

Mutations Y30A and Y196A (amino acid numbering corresponds to prototoxin without the 13 amino acids N-terminal peptide sequence) were introduced into the gene encoding epsilon prototoxin (P-Etx) using the QuickChange Lightning Site-Directed Mutagenesis Kit (Agilent Technologies, Inc. Santa Clara, US) according to the manufacturer's instructions. Recombinant P-Etx with Y30A and Y196A mutations is termed Y30A-Y196A.

Recombinant Y30A-Y196A was expressed, purified and its thermostability assessed as described previously [14].

### 2.2 Trypsin activation

Purified recombinant Etx prototoxin was activated with trypsin, TPCK treated from bovine pancreas (Sigma-Aldrich Company Ltd., Gillingham, UK) for 1 h at room temperature and removal of the C-terminal peptide sequence was assessed by SDS-PAGE as described previously [14].

### 2.3 Cell culture

MDCK.2 cells (ATCC-LGC Standards, Teddington, UK) and ACHN cells (ECACC, Salisbury, UK) were routinely cultured in Eagle's Minimum Essential Medium (EMEM; ATCC-LGC Standards, Teddington, UK) supplemented with 10% Foetal Bovine Serum Gold (PAA, Pasching, Austria) at 37 °C in a humidified atmosphere of 95% air/5% CO_2_. The culture medium was replaced every 2–3 days. Cells were routinely detached by incubation in trypsin/EDTA and split as appropriate (typically 1:6 dilutions).

### 2.4 Cytotoxicity assay

The cytotoxic activity of trypsin-activated toxin toward MDCK.2 and ACHN cells was determined by measuring the amount of lactate dehydrogenase (LDH) released from the cytosol of lysed cells into the cell culture medium using the CytoTox 96 nonradioactive cytotoxicity assay kit (Promega UK, Southampton, UK) as described previously [14]. The toxin dose required to kill 50% of the cell monolayer (CT_50_) was determined by nonlinear regression analysis using GraphPad Prism 6 software (GraphPad Software, La Jolla, USA). All experiments were performed in triplicate with three technical replicates each.

### 2.5 On-Cell Western assay

To measure binding of prototoxin to MDCK.2 and ACHN cells the On-Cell Western assay was used as described previously [14]. Bound prototoxin was detected with mouse anti-Etx monoclonal Bio355 antibody (Bio-X Diagnostics S.P.R.L, Belgium) and IRDye 800CW goat anti-mouse IgG (H + L) antibody (LI-COR Biosciences, Lincoln, USA) at 1:500 dilution each. To quantify the amount of fluorescent signal, plates were imaged at 800 nm using the Odyssey CLx infrared imaging system (LI-COR Biosciences, Lincoln, USA). The binding activity of the mutant prototoxin was expressed as the percentage of fluorescence intensity relative to wild type prototoxin. To compare the means of the On-Cell Western assay data, Two-Way ANOVA analysis followed by Dunnett's multiple comparisons test was carried out using the GraphPad Prism 6 software (GraphPad Software, La Jolla).

### 2.6 Immunisation of rabbits with recombinant Y30A-Y196A prototoxin

A group of three New Zealand White rabbits were immunised subcutaneously eight times on days 0, 14, 28, 42, 56, 70, 84 and 98 with a dose of 200 μg purified recombinant Y30A-Y196A prototoxin in phosphate buffered saline, pH 7.2 (PBS) (Immune Systems Ltd., UK). For the initial immunisation Freund's complete adjuvant was used. The remainder immunisations used Freund's incomplete adjuvant. Pre-immune sera were collected on day 0 and harvest bleed was collected on day 107. Post-inject antibodies were detected by indirect ELISA (Immune Systems Ltd., UK). In brief, a two-fold dilution series of each serum (ranging from 1:100 to 1:204,800) was prepared and added to a 96-well plate coated with recombinant Y30A-Y196A prototoxin. A horseradish-peroxidase-conjugated immunoglobulin antibody (IgG-HRP) was used to detect bound antibody and plates were developed by the addition of ABTS substrate. Titres were calculated by measuring the dilution point where the absorbance at OD_405nm_ dropped below 0.2 (4 times background).

### 2.7 *In vitro* neutralisation assay

Trypsin-activated wild type Etx at a dose of 1× CT_50_ was incubated for 1 h at room temperature with serial dilutions of either Y30A-Y196A rabbit polyclonal antiserum or with a negative control antibody. The toxin-antibody mixtures were added to MDCK.2 cells plated in a 96-well plate and incubated at 37 °C for 3 h before cytotoxicity was measured by the LDH assay as described above. Data were expressed relative to the LDH released from cells treated with toxin only.

### 2.8 Toxicity of trypsin activated Y30A-Y196A in mice

Groups of six female BALB/c mice were challenged by the intraperitoneal route with a dose of trypsin-activated wild type toxin corresponding to 1×, 10×, 100× or 1000× the expected LD_50_ dose of wild type toxin in phosphate buffered saline, pH 7.2 (PBS) (2 ng, 20 ng, 200 ng or 2 μg/mouse, respectively, in 100 μl volume) or with a dose of trypsin-activated Y30A-Y196A corresponding to 10× or 1000× the expected LD_50_ dose of wild type toxin in PBS (20 ng or 2 μg/mouse, respectively, in 100 μl volume). The amounts of trypsin-activated toxins used in this study are listed in Supplementary Table 1. Control animals received 100 μl PBS each. The challenged animals were monitored continuously for the first hour post challenge, at hourly intervals until 6 h post challenge and then at further 6 h intervals. The experiment was terminated at 24 h post challenge.

The challenged animals were monitored continuously and scored according to severity of clinical signs and neurological effects on a scale of 0–3, with 0 indicating no change and values between 1 and 3 indicating increasing severity. Details of the scoring system are described in Supplementary Table 2. The onset of neurological symptoms marked a humane endpoint and animals showing neurological symptoms were euthanized. The use of animals was conducted in accordance with the Animals (Scientific Procedures) Act (1986) and was performed with the approval of the on-site animal ethics committee.

## 3 Results

### 3.1 Identification of recombinant Y30A-Y196A as a vaccine candidate

**Fig. 1:**
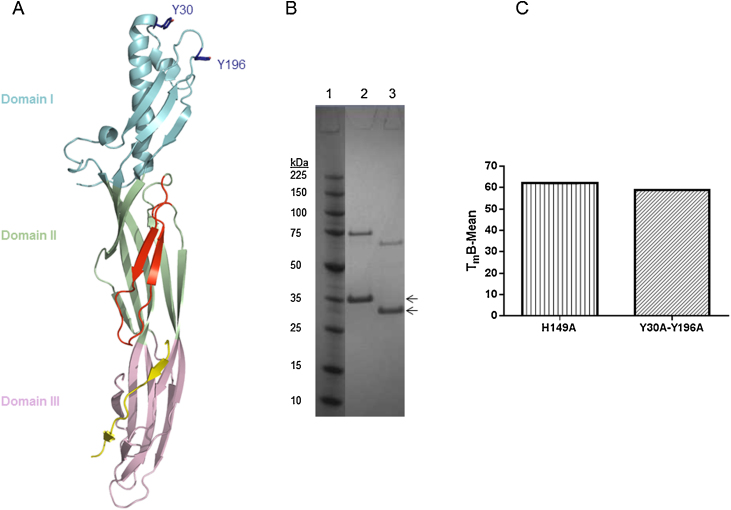
Recombinant Etx mutant Y30A-Y196A. (A) Ribbon representation of recombinant epsilon prototoxin with side chains of amino acids Y30 and Y196 in Domain I shown in stick representation. Amino acid numbering corresponds to prototoxin without the 13 amino acids N-terminal peptide sequence (PDB ID: 1UYJ). (B) SDS-PAGE analysis of purified Y30A-Y196A. Lane 1: Perfect Protein marker, molecular mass is indicated in kDa to the left; lane 2: purified Y30A-Y196A prototoxin; Lane 3: trypsin activated Y30A-Y196A. Proteins were visualized by Coomassie staining. Arrows indicate the positions of inactive prototoxin and trypsin activated toxin, respectively. (C) Thermostability of Y30A-Y196A prototoxin was determined by the Boltzmann method using the Protein Thermal Shift software (Applied Biosystems). Results represent the mean and standard deviation of triplicate samples.

In an attempt to identify an Etx variant with markedly reduced toxicity relative to wild type toxin that could be considered as a recombinant vaccine candidate, we combined mutations Y30A and Y196A, generating the double tyrosine mutant, termed Y30A-Y196A (Fig. 1A). Etx mutant Y30A-Y196A was expressed and purified as described in Materials and Methods. Purified recombinant Y30A-Y196A prototoxin had an apparent molecular weight of ∼37 kDa as detected by SDS-PAGE (Fig. 1B, lane 2). Thermal stability assay [16] revealed that the melting temperature (*T*_m_) of Y30A-Y196A was similar to that of Etx with H149A mutation, providing further evidence that the double tyrosine mutant is folded correctly (Fig. 1C). The H149A mutation has previously been shown not to have an effect on the prototoxin tertiary structure [14].

### 3.2 Cytotoxic activity of trypsin activated Y30A-Y196A toward MDCK.2 and ACHN cells

**Fig. 2:**
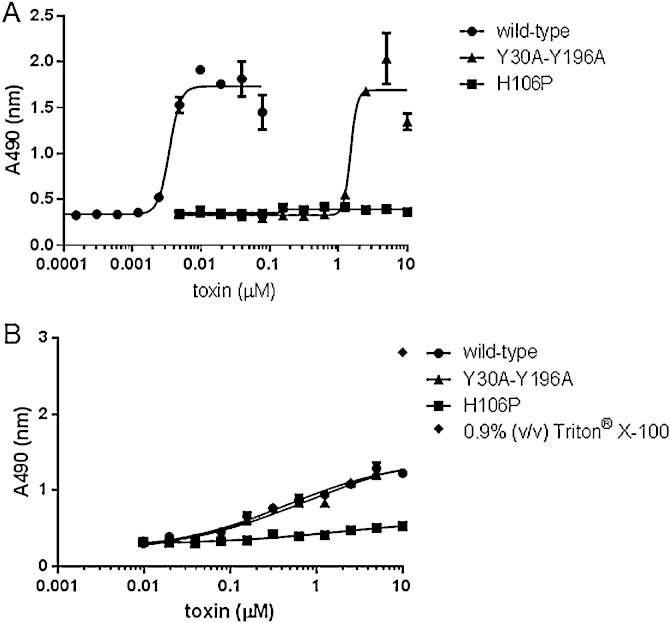
Cytotoxicity of trypsin activated recombinant Y30A-Y196A toward MDCK.2 and ACHN cells. A two-fold dilution series (ranging from 10 μM to 0.15 nM) of each activated toxin was added to (A) MDCK.2 or (B) ACHN cells seeded into a 96-well plate. After 3 h incubation at 37 °C, the cell culture medium was harvested and cytotoxicity was measured using the CytoTox 96 nonradioactive cytotoxicity assay kit (Promega).

The cytotoxic activity of trypsin activated Y30A-Y196A toward MDCK.2 and ACHN cells were measured by the LDH assay. The average dose of Y30A-Y196A required to kill 50% of MDCK.2 cells was determined to be 1.49 μM, corresponding to an approximately 430-fold reduction in cytotoxic activity relative to wild type Etx with a CT_50_ value of 3.47 nM (Fig. 2A). In contrast, the results of our cytotoxicity assay in ACHN cells revealed that the cytotoxic activity of trypsin activated Y30A-Y196A was equivalent to that of wild type toxin (Fig. 2B). No LDH release could be measured when MDCK.2 or ACHN cells were treated with trypsin activated Etx mutant H106P [17], even at the maximum concentration of 10 μM tested.

### 3.3 Binding of Y30A-Y196A prototoxin to MDCK.2 and ACHN cells

**Fig. 3:**
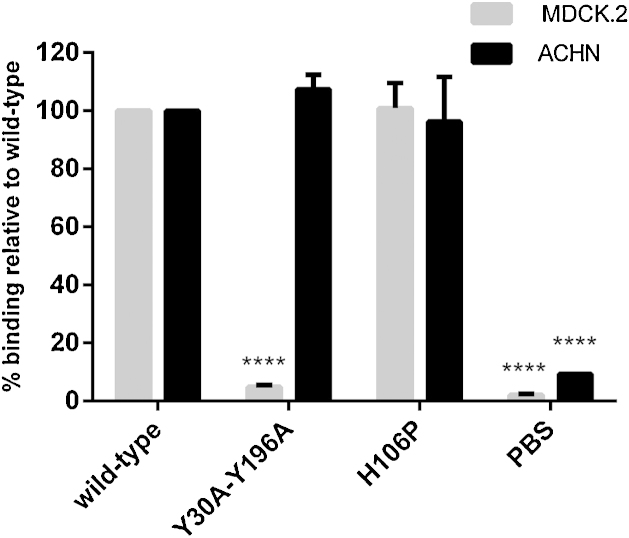
Binding of Y30A-Y196A prototoxin to MDCK.2 and ACHN cells. Purified recombinant prototoxins (5 μM) were added to wells containing MDCK.2 or ACHN cells. After 20 min incubation at 37 °C, cells were fixed and bound protein was detected with mouse anti-Etx and IRDye 800CW goat anti-mouse antibodies. Asterisks denote a statistically significant difference (^****^p < 0.0001; two-way ANOVA analysis) relative to wild type control. Each bar represents the means ± SEM of three independent experiments performed in triplicate.

We also evaluated the effect of Y30A-Y196A prototoxin on its ability to bind to MDCK.2 and ACHN cells using the On-Cell Western assay. As shown in Fig. 3, the fluorescent signal of MDCK.2 cells treated with Y30A-Y196A prototoxin was similar to that of cells treated with PBS only. In contrast, ACHN cells treated with Y30A-Y196A prototoxin showed fluorescence equivalent to that of cells treated with wild type toxin (Fig. 3). Etx mutant H106P showed similar binding to wild type toxin in both cell lines (Fig. 3).

### 3.4 Y30A-Y196A polyclonal antiserum raised in rabbits is able to induce protective immunity *in vitro*

The mean IgG titre against purified Y30A-Y196A prototoxin was measured by indirect ELISA on day 107 of the immunisation schedule and determined to be 1:16,000 (Immune Systems Ltd., UK), indicating that immunisation of rabbits with Y30A-Y196A prototoxin induced a specific antibody response.

**Fig. 4:**
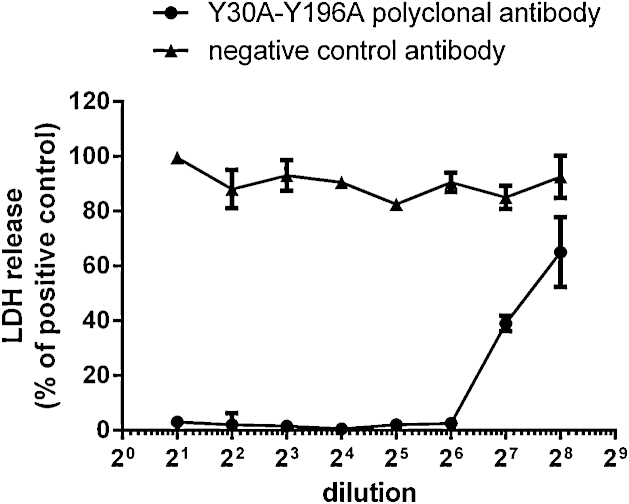
*In vitro* neutralisation assay. The polyclonal rabbit antiserum raised against Y30A-Y196A prototoxin is able to protect MDCK.2 cells against wild type Etx-induced cytotoxicity in a dose-dependent manner (up to dilution 2^6^, which corresponds to 0.195 μg/ml antibody concentration).

To test the ability of the polyclonal antiserum raised in rabbits against Y30A-Y196A prototoxin to neutralise the cytotoxic activity of wild type Etx in MDCK.2 cells, we used the *in vitro* neutralisation assay as described in Materials and Methods. As shown in Fig. 4, the polyclonal antiserum raised against Y30A-Y196A prototoxin was able to protect MDCK.2 cells against wild type Etx-induced cytotoxicity in a dose-dependent manner (up to dilution 2^6^, which corresponds to 0.2 μg/ml antibody concentration). In contrast, the negative control antibody did not inhibit Etx-induced cytotoxicity at any of the doses tested.

### 3.5 Toxicity of Y30A-Y196A in mice

Our *in vitro* toxicity data revealed that trypsin activated Y30A-Y196A has differential cytototoxic activity toward MDCK.2 and ACHN cells, showing markedly reduced cytotoxicity in MDCK.2 cells but equivalent cytotoxic activity to wild type toxin in ACHN cells. Therefore, we next tested the toxicity of trypsin activated Y30A-Y196A after intraperitoneal administration in groups of six mice.

First, we determined the toxicity of trypsin activated wild type Etx after intraperitoneal administration in groups of six mice. Mice injected with 1× and 10× LD_50_ of wild type toxin survived for 24 h without showing any signs of intoxication, whereas a dose of 100× LD_50_ resulted in death within 180 min post-injection and a dose of 1000× LD_50_ resulted in death by 45.5 min post-injection.

**Fig. 5:**
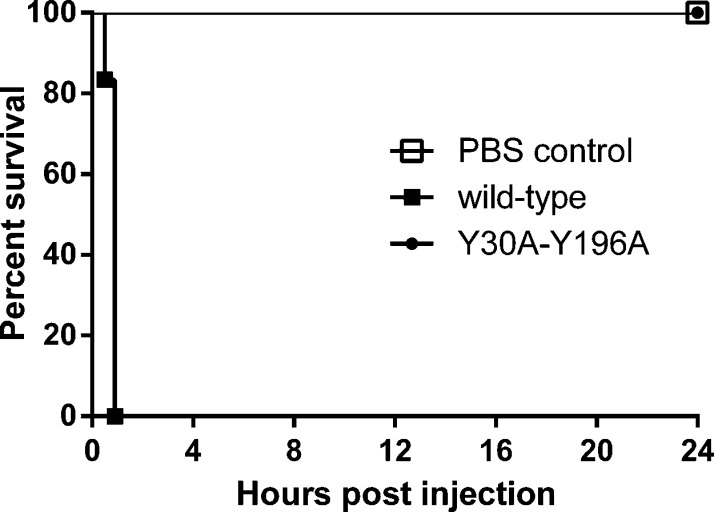
Toxicity of trypsin activated Y30A-Y196A after intraperitoneal injection of mice. (A) Kaplan-Meier survival curves of female BALB/c mice over a 24 h period following intraperitoneal injection of 1000× LD_50_ dose of trypsin-activated wild type or Y30A-Y196A toxins. Control animals received PBS only. Animals showing neurological signs were euthanized at a humane endpoint.

To test the toxicity of Y30A-Y196A *in vivo*, mice were injected with trypsin activated Y30A-Y196A at a dose of 1000× LD_50_ of trypsin-activated wild type toxin. Control animals received PBS only. As shown in Fig. 5A, mice injected with either PBS or Y30A-Y196A survived for 24 h without showing any signs of intoxication, while mice injected with wild type toxin died within 50 min.

## 4 Discussion

Recently, we have determined the roles of surface exposed tyrosine residues in domain I of Etx mediating binding and toxicity of Etx to target cells [14]. This study was conducted to determine the potential of the site-directed Etx mutant Y30A-Y196A to be exploited as a recombinant vaccine against enterotoxemia.

Site-directed mutants of Etx with markedly reduced toxicity have previously been produced [17,18]. The site-directed mutant H106P with no activity has been shown to be non-toxic to mice after intravenous administration of periplasmic extracts from *Escherichia coli* [17]. Moreover, immunisation of mice with H106P mutant resulted in the induction of a specific antibody response and immunised mice were protected against a subsequent challenge of 1000× LD_50_ dose of wild type Etx administered by the intravenous route [17]. The low toxicity site-directed Etx mutant F199E has recently been shown to protect immunised mice against a 100× LD_50_ dose of recombinant wild type Etx toxin [18]. While these Etx mutants are promising vaccine candidates against enterotoxemia, recombinant Etx vaccines derived from site-directed mutants with a single mutation risk reversion to full activity in a DNA based vaccine or in a live vaccine vector such as *Salmonella*. Therefore, the use of recombinant Etx vaccines derived from low toxicity site-directed mutants with two mutations, such as the Y30A-Y196A mutant developed in this study, would greatly reduce the risk of reversion to full activity, making Y30A-Y196A an ideal recombinant vaccine candidate.

Simultaneous replacement of Y30 and Y196 with alanine generated a stable variant of Etx that showed significantly reduced cell binding and cytotoxic activities in MDCK.2 cells but not in ACHN cells. Single mutants Y30A and Y196A have previously been shown to have 27-fold and 10-fold reduction in cytotoxicity toward MDCK.2 cells relative to Etx-H149A (with an average CT_50_ of 12 nM), respectively [14], which corresponds to 93-fold and 34-fold reduction in cytotoxic activity relative to wild type Etx (with an average CT_50_ value of 3.47 nM), respectively. Mutant Y30A-Y196A in this study showed 430-fold reduction in cytotoxic activity relative to wild type Etx in MDCK.2 cells, suggesting that mutations Y30A and Y196A have a cumulative effect on reducing the ability of Etx to lyse MDCK.2 cells. In contrast, the double mutant Y30A-Y196A showed no reduction in cytotoxic activity in ACHN cells relative to wild type toxin, further supporting the findings of our previous study that surface exposed tyrosine residues in domain I do not mediate cytotoxicity of Etx in ACHN cells [14]. These data suggest that Etx may have a dual mechanism of binding to target cells, similar to *Staphylococcus aureus* alpha hemolysin (α-HL) [19].

Due to the differential activity of mutant Y30A-Y196A in MDCK.2 and ACHN cells, we assessed the safety of this variant for immunisation by intraperitoneal administration of trypsin activated Y30A-Y196A to mice. There is a scarcity of data on the LD_50_ dose of Etx in the literature when given by the intraperitoneal route to mice. Thus, this study also determined the toxicity of trypsin activated wild type Etx after intraperitoneal administration in groups of six mice. In previous studies trypsin activated Etx has been shown to have a LD_50_ dose ranging from 70 ng/kg [20] to 320 ng/kg [10] when administered by the intravenous route to mice. There is less data on the LD_50_ dose of wild type Etx when given by the intraperitoneal route to mice. Intraperitoneal injection of Etx prototoxin into Fisher rats with an average weight of 350 g produced a LD_50_ of 14 μg/animal or 40 μg/kg of body weight [21]. Taking into account that Etx prototoxin is >1000-fold less active compared to activated toxin [22], intraperitoneal injection of activated Etx would yield a LD_50_ of approximately 40 ng/kg of body weight. This figure correlates well with the consensus LD_50_ value of 100 ng/kg after intravenous administration of activated Etx to mice [23]. Therefore, our working assumption was that the LD_50_ value of trypsin activated wild type Etx after intraperitoneal administration to mice is 100 ng/kg of body weight or approximately 2 ng/mouse with an average weight of 20 g. Mice injected with 2 ng or 20 ng trypsin activated wild type Etx by the intraperitoneal route survived for 24 h without showing any signs of intoxication, whereas a dose of 200 ng trypsin activated wild type Etx resulted in death within 180 min post-injection, suggesting that the LD_50_ value of trypsin activated wild type Etx administered to mice by the intraperitoneal route is between 20 ng and 200 ng/mouse, extrapolated to 1–10 μg/kg of body weight.

We showed that Y30A-Y196A is inactive in mice after intraperitoneal administration of up to 1000× the expected LD_50_ dose of wild type toxin, mirroring our *in vitro* cytotoxicity data in MDCK.2 cells. We also showed that polyclonal antibody raised against Y30A-Y196A provides protection against wild type toxin in an *in vitro* neutralisation assay.

In conclusion, this study showed that recombinant Etx mutant Y30A-Y196A is non-toxic to mice, demonstrating the potential of Y30A-Y196A mutant to form the basis of an improved recombinant vaccine against enterotoxemia in ruminants. Further studies are needed to determine whether Y30A-Y196A is able to induce protection against experimental enterotoxemia in sheep.

## Authors’ contributions

MBB and CAH carried out most of the experiments and drafted the manuscript. CAH carried out and CV assisted with the *in vivo* toxicity assay. SPFC, CGS, CEN and ARC helped with experiments and interpreted the data. RT, DSM and AKB designed research and revised the manuscript. All authors read and approved the final manuscript.

## Conflict of interest statement

The authors have no competing interests.
