# Supplementary material for: Clostridium perfringens epsilon toxin mutant Y30A-Y196A as a recombinant vaccine candidate against enterotoxemia
Source: Vaccine. 2014 May 13;32(23):2682–7. doi: 10.1016/j.vaccine.2014.03.079 (PMC4022833; doi:10.1016/j.vaccine.2014.03.079)
Supplement: Supplementary file 1 [file mmc1.docx]

# Supplementary Table 1. The amounts of trypsin-activated toxins used in this study.

| **Dose** | **Amount / mouse** | **Concentration** | **Volume dosed** |
| --- | --- | --- | --- |
| Wild type | | | |
| 1 x LD_50_ | 2 ng | 20 ng/ml | 100 μl / mouse |
| 10 x LD_50_ | 20 ng | 200 ng/ml | 100 μl / mouse |
| 100 x LD_50_ | 200 ng | 2 μg/ml | 100 μl / mouse |
| 1000 x LD_50_ | 2 μg | 20 μg/ml | 100 μl / mouse |
| Y30A-Y196A | | | |
| 10 x LD_50_ | 20 ng | 200 ng/ml | 100 μl / mouse |
| 1000 x LD_50_ | 2 μg | 20 μg/ml | 100 μl / mouse |
