# Supplementary material for: Clostridium perfringens epsilon toxin mutant Y30A-Y196A as a recombinant vaccine candidate against enterotoxemia
Source: Vaccine. 2014 May 13;32(23):2682–7. doi: 10.1016/j.vaccine.2014.03.079 (PMC4022833; doi:10.1016/j.vaccine.2014.03.079)
Supplement: Supplementary file 2 [file mmc2.docx]

# Supplementary Table 2. Summary of severity scoring system. Groups of six mice were administered trypsin activated wild type Etx or Etx mutant Y30A-Y196A by the intraperitoneal route and were scored on general clinical signs and neurological symptoms. An overall severity score was determined by averaging the individual scores for these two parameters.

| **General clinical signs** | |
| --- | --- |
| Severity score | Symptoms |
| 0 | Normal |
| 1 | Ruffled fur/rapid breathing |
| 2 | Ruffled fur, less mobile, isolated |
| 3 | Above and hunched appearance/restless or very still/not alert |
| **Neurological signs** | |
| Severity score | Symptoms |
| 0 | Normal |
| 1 | Loss of righting reflex/tail paralysis/incomplete hind limb paralysis/incoordination |
| 2 | Involuntary limb movements/complete hind limb paralysis |
| 3 | Full body paralysis/moribund |
